# Rapid and sensitive detection of superoxide dismutase in serum of the cervical cancer by 4-aminothiophenol-functionalized bimetallic Au-Ag nanoboxs array

**DOI:** 10.3389/fbioe.2023.1111866

**Published:** 2023-03-02

**Authors:** Ji Xia, Gao-Yang Chen, You You Li, Lu Chen, Dan Lu

**Affiliations:** ^1^ Institute of Translational Medicine, Medical College, Yangzhou University, Yangzhou, China; ^2^ Department of Oncology, The Second People’s Hospital of Taizhou City, Taizhou, China; ^3^ Jiangsu Key Laboratory of Integrated Traditional Chinese and Western Medicine for Prevention and Treatment of Senile Diseases, Yangzhou University, Yangzhou, China

**Keywords:** surface-enhanced Raman scattering, 4-aminothiophenol, dithiol azobenzene, Au-Ag nanoboxs, cervical cancer

## Abstract

Early, efficient and sensitive detection of serum markers in cervical cancer is very important for the treatment and prognosis to cervical cancer patients. In this paper, a SERS platform based on surface enhanced Raman scattering technology was proposed to quantitatively detect superoxide dismutase in serum of cervical cancer patients. Au-Ag nanoboxs array was made by oil-water interface self-assembly method as the trapping substrate. The single-layer Au-AgNBs array was verified by SERS for possessing excellent uniformity, selectivity and reproducibility. 4-aminothiophenol (4-ATP) was used as Raman signal molecule, it will be oxidized to dithiol azobenzene under the surface catalytic reaction with the condition of PH = 9 and laser irradiation. The quantitative detection of SOD could be achieved by calculating the change of characteristic peak ratio. When the concentration was from 10 U mL^−1^–160 U mL^−1^, the concentration of SOD could be accurately and quantitatively detected in human serum. The whole test was completed within 20 min and the limit of quantitation was 10 U mL^−1^. In addition, serum samples from the cervical cancer, the cervical intraepithelial neoplasia and healthy people were tested by the platform and the results were consistent with those of ELISA. The platform has great potential as a tool for early clinical screening of cervical cancer in the future.

## Introduction

Among female malignancies, cervical cancer is the second leading cause of death in women, with more than 600,000 new cases each year, accounting for 5% of all new cancer cases, more than 80% of which occur in developing countries (Bray et al., 2018; Siegel et al., 2018; Clarke et al., 2019; Liu et al., 2019). There are even 300,000 deaths ever year and a clear trend of younger age (Li et al., 2011; Arbyn et al., 2022). Squamous cell carcinoma is the most common histological type of cervical cancer and persistent infection of HPV is the main cause of cervical squamous cell carcinoma (Li et al., 2016; Mao et al., 2018). The current diagnostic methods for cervical cancer include HPV testing, cytology testing and colposcopy, but these methods are usually invasive and have low patient acceptance. Although the popularity of vaccines and cervical cancer screening has effectively reduced the mortality rate of cervical cancer. Cervical cancer is still the malignant tumor with the highest mortality rate in women worldwide (Aggarwal, 2014). However, the early stage of cervical cancer is not easy to diagnose and existing diagnostic methods are inaccurate and expensive (Zorzi et al., 2013; Tsikouras et al., 2016). Therefore, a sensitive and non-invasive method for diagnosing cervical cancer is urgently needed. With the development of tumor ecology, biomarkers have gradually become one of the indicators for early detection and prognosis of tumors (Braham et al., 2017).

Superoxide dismutase is the most common class of antioxidant enzymes in organisms and it is also an important enzyme that regulates the metabolism of reactive oxygen species. SOD achieves cellular homeostasis by maintaining intracellular reactive oxygen species levels and redox balance, while protecting normal tissues from oxidative stress. Studies have shown that the downregulation of SOD activity is related to tumorigenesis and development. The reduction of SOD levels may lead to an increase in lipid peroxidation, resulting in rigidity and deformability of cells, which may be related to tumor migration and invasion (Khawsak et al., 2012). The SOD activity in normal human blood is about 128 U mL^−1^, while the SOD activity in tumor patients is significantly lower than that in normal people. For example, the SOD activity in the serum of gastric carcinoma is 27.6 ± 6.6 U mL^−1^ (Naidu et al., 2007), while SOD activity in the serum of patients with intestinal cancer is 79.35 ± 15.66 U mL^−1^ (Lee et al., 2006). Therefore, rapid and simple detection of SOD concentration is of great significance for early diagnosis of tumor. At present, there are three commonly used methods to detect SOD activity, including nitroblue tetrazole photochemical reduction method, chemiluminescence method and pyrogallol autoxidation method. Nitroblue tetrazole photochemical reduction method has strong specificity, stable determination results, good repeatability, simple instrument, but it also has complicated reagent preparation, complex operation, long determination time and expensive reagents (Manoharan et al., 2004; Qi et al., 2022). Chemiluminescence method has the advantages of high sensitivity, high accuracy, strong specificity detection. However, due to the need for special high sensitivity precision luminescence detection instruments, its clinical use is inhibited. The pyrogallol autoxidation method requires high environmental conditions and is not easy to implement. In clinical detection, turbidimetry, electron spin resonance (ESR) spectroscopy and spectrophotometry were often used for the analysis of SOD (Manju et al., 2002; Zahra et al., 2021). These methods take a long time to detect, expensive and not easy to popularize, hence, it is urgent to develop a rapid, sensitive and cheap method to detect SOD.

Surface-enhanced Raman scattering is a convenient, non-destructive and ultra-sensitive molecular fingerprinting spectroscopy method, which has been widely used in chemical, biological and food fields (Wang et al., 2018; Yang et al., 2019; Zhai et al., 2021). Theoretically, SERS is mainly based on the interaction of incident laser light with nanostructures, resulting in electromagnetic field enhancement in nanostructure gaps or junctions (hot spots). The enhancement factor depends on the size, shape, distribution and material composition of the nanostructures (Mondal and Subramaniam, 2020; Fu et al., 2021). The analyte molecules close to the hot spot region help to generate stronger Raman signals. SERS can overcome the disadvantage of low Raman spectral sensitivity and greatly expand the Raman signal. Its narrow linewidth allows the detection of multiple analytes in complex mixtures. The surface selection rule and the selectivity of resonance enhancement enable SERS to enhance only target molecules or chemical groups in extremely complex systems to obtain the spectral information of target analytes (Jiang et al., 2018; Luu et al., 2020).

Compared with common nano particles, such as gold nano particles and gold nano stars, Au-Ag nanoboxs (Au-AgNBs) with regular appearance is gradually attracting attention. Due to the inner and transmural walls of the cavity, Au-AgNBs have superior coupled electromagnetic fields between the inner and outer walls due to the coupling of the inner and outer surface fields, resulting in strong light absorption and high Raman enhancement (Xiong et al., 2005; Mahmoud et al., 2012). The pores on the surface of Au-AgNBs are expected to promote SERS activity through large electric field enhancement. In addition, due to the large surface area of Au-AgNBs, more Raman signal molecules can be accommodated on the surface to enhance the sensitivity of SERS detection (Wang et al., 2019).

High performance SERS substrate is a key problem in the application of SERS technology. Its signal strength is related to the surface morphology of the substrate adsorbed by the molecules. By assembling different kinds of nanomaterials into the substrate. The assembled substrates are divided into disordered substrates and highly ordered substrates and their repeatability is another important factor in the quantitative detection of target substances. Compared with the disordered SERS substrate, the highly ordered SERS substrate guarantees the reliability of the data due to its excellent signal uniformity and repeatability (Cheng et al., 2020; Langer et al., 2020; Yun and Koh, 2020). Au-AgNBs have the advantages of mild reaction conditions, simple steps, uniform morphology and high biocompatibility. Assembling them into orderly SERS substrates can not only increase the density of hot spots, but also enhance the SERS effect activity (Sultangaziyev and Bukasov, 2020).

**SCHEME 1:**
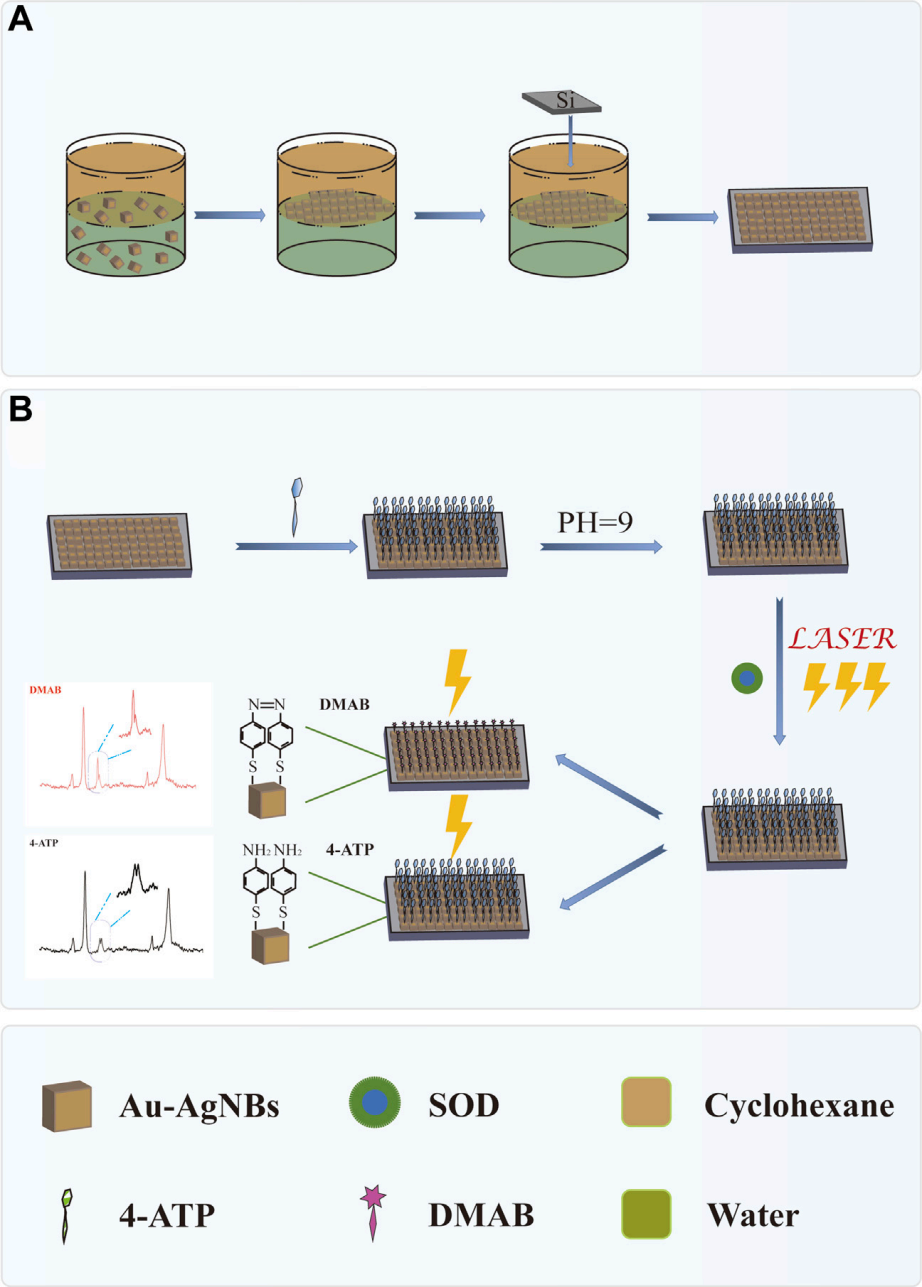
**(A)** Preparation of self-assembled Au-AgNBs array at oil-water interface. **(B)** SOD was detected by SERS platform.

In this work, a novel SERS platform for SOD detection based on Au-AgNBs array was proposed. 4-aminothiophene (4-ATP) was used as Raman reporter, which had been widely studied and used to determine SERS capability. Scheme 1 showed the schematic diagram of SERS platform preparation and SOD detection. By means of oil-water interface self-assembly, Au-AgNBs was assembled on the substrate surface of silicon wafer to form Au-AgNBs ordered nanoarray. The homogeneity, sensitivity and stability of the array were tested. Under the condition of PH = 9 and laser irradiation, with the gradual decrease of SOD concentration, 4-ATP would be oxidized into dithiol azobenzene (DMAB) under the action of surface catalytic reaction driven by plasmon, which made SERS signal appear the characteristic peak of DMAB and the SERS signal intensity changed accordingly, so as to realize the qualitative and quantitative detection of SOD. Finally, the SERS platform was used to detect SOD in clinical specimens of healthy people, patients with cervical low-grade squamous intraepithelial disease (LSIL), high-grade squamous intraepithelial disease (HSIL) and cervical cancer. The ELISA results further verified the accuracy of the method. This method had great potential in the early screening of cervical cancer.

## Materials and methods

### Materials

Chloroauric acid tetrahydrate (HAuCl_4_-4H_2_O), silver nitrate (AgNO_3_), hydrogen peroxide, sulfuric acid and ascorbic acid (AA) were obtained from YangZhou LanTian Chemicals Co. Ltd. (China). 4-aminothiophenol (4-ATP), sodium chloride and ethanol were acquired from Jiangsu Younuo Chemicals Co. Ltd. (China). All of the materials were applied directly without further processing. Meanwhile, ELISA kits, superoxide dismutase and four necked round bottom flasks were all purchased from Sangon Biotech (Shanghai, China). Deionized water (resistivity >18.2 Ω) was used for the preparation of the specimens and throughout all the experiments. All glassware was dipped in aqua regia [HNO_3_/HCl = 1:3 (v/v)] for over 24 h and washed with deionized water.

### Collection, treatment, and preservation of clinical serum samples

Peripheral blood samples from clinical medical college of Yangzhou university in 50 cases of healthy subjects and 50 patients with low grade squamous intraepithelial lesion, high-grade squamous intraepithelial lesion in 50 cases of patients and 50 cases of cervical cancer patients were centrifuged at 3,000 rpm for 12 min at 4°C. Then serum samples were collected and based on its classification to store in −80°C before analysis. Consent documents were obtained from all donors. Table 1 summarizes the details of age and histopathological stage.

**TABLE 1:** Basic characteristics of the subjects to be included.

Groups	Healthy person	LSIL	HSIL	Cervical cancer
Age (mean)	29	38	42	48
Sample	50	50	50	50

### Synthesis of Au-AgNBs

Au-AgNBs modified by nanodots was synthesized by one-step method (Li et al., 2018). The experiment was carried in 100 mL conical tubes. First, 90 μL of 1% HAuCl_4_ solution was added into 10 mL ultrapure water by continuously stirring for 1 min. Then, 170 μL of AgNO_3_ (6 mM) was dropped into the mixture and the mixture became turbid pearl white. After dropping the 125 μL of AA (0.1 M), the solution turned to distinct blueviolet color indicating the Au-AgNBs were prepared. After 10 min of continuously stirring, the Au-AgNBs were concentrated by centrifugation (4,000 rpm, 4 min). Collecting the bottom sediment, disperse the particles in 5 mL deionized water and store at 4°C.

### Manufacturing of capture substrate

The silicon wafer was divided with the size of 0.8 × 0.8 cm^2^, then they were placed in a beaker and used after ultrasonic cleaning with ultra-pure water and ethanol in turn. Then, the silicon wafer of appropriate size was immersed in piranha solution (hydrogen peroxide (30%) was added to concentrated sulfuric acid in a ratio of 3:7 by volume) for 30 min to make the silicon wafer hydrophilic. Then ultrapure water and ethanol were used again to clean the silicon wafer for three times. Au-AgNBs prepared the array by using the method of oil-water interface self-assembly. In brief, by mixing 8 mL of the prepared Au-AgNBs solution sequentially with 4 mL of hexane in a beaker and then adding 4 mL of ethanol drop by drop, it was found that Au-AgNBs formed neat arrays at the oil-water interface. Next, the Au-AgNBs array was picked up using the prepared hydrophilic silicon wafer and placed in a ventilated place to dry. Then, the Au-AgNBs array is obtained. These prepared Au-AgNBs array were uniformly stored in a sealed glass cover at 4°C. Every time they were used, Au-AgNBs arrays made in the same batch were selected to reduce the error caused by the detection platform.

### Principle of the SERS platform

Before the test, 20 μL of 2.5 × 10^−5^ M 4-ATP was dropped onto the prepared Au-AgNBs array surface. Then an appropriate amount of buffer solution was added to the array surface to adjust PH = 9 and left it at room temperature for 2 min to mix evenly. Next, 20 μL of sample solution was dropped and stayed for 2 min at room temperature to make it evenly covered. The ordered array was irradiated by 785 nm laser. The laser power at the sample location was 2.3 mW. Under the continuous irradiation of the sample, the SERS spectrum of 1 s was continuously measured in 1 min steps. All SERS spectrum reported in this study were collected in a continuous mode within the range of 400–1800 cm^−1^. The average SERS spectra measured at 10 different points at random in one platform were used to quantify SOD in the sample solution, which ensured the authenticity and rationality of the data. The characteristic bands of 4-ATP and DMAB were listed in the table S1 (supporting information).

### Instrumentation

Uv-vis-near-infrared (UV-VIS-NIR) spectrometer (UV-3000PC, Mapuda, China) was used to detect the absorption spectrum of UV-VIS-NIR. Transmission electron microscope (TEM) images were taken with transmission electron microscope (Tecnai 12, Philips, Netherlands). Scanning electron microscopy (SEM) images were studied using an S-4800II ⅐ laser emission scanning electron microscope (Gemini SEM 300, Carl Zeiss, Germany). High-resolution TEM (HRTEM) images, selective region electron diffraction (SAED) and element mapping images were obtained using a field emission transmission electron microscope (Tecnai G2F30 S-Twin, FEI, United States). Raman spectrometer (Renishaw inVia, United Kingdom) was used to record SERS mapping with a mapping step of 1 μm and a pinhole of 25 μm. All experiments were performed at room temperature.

## Results and discussion

### Characterization of Au-AgNBs

**FIGURE 1:**
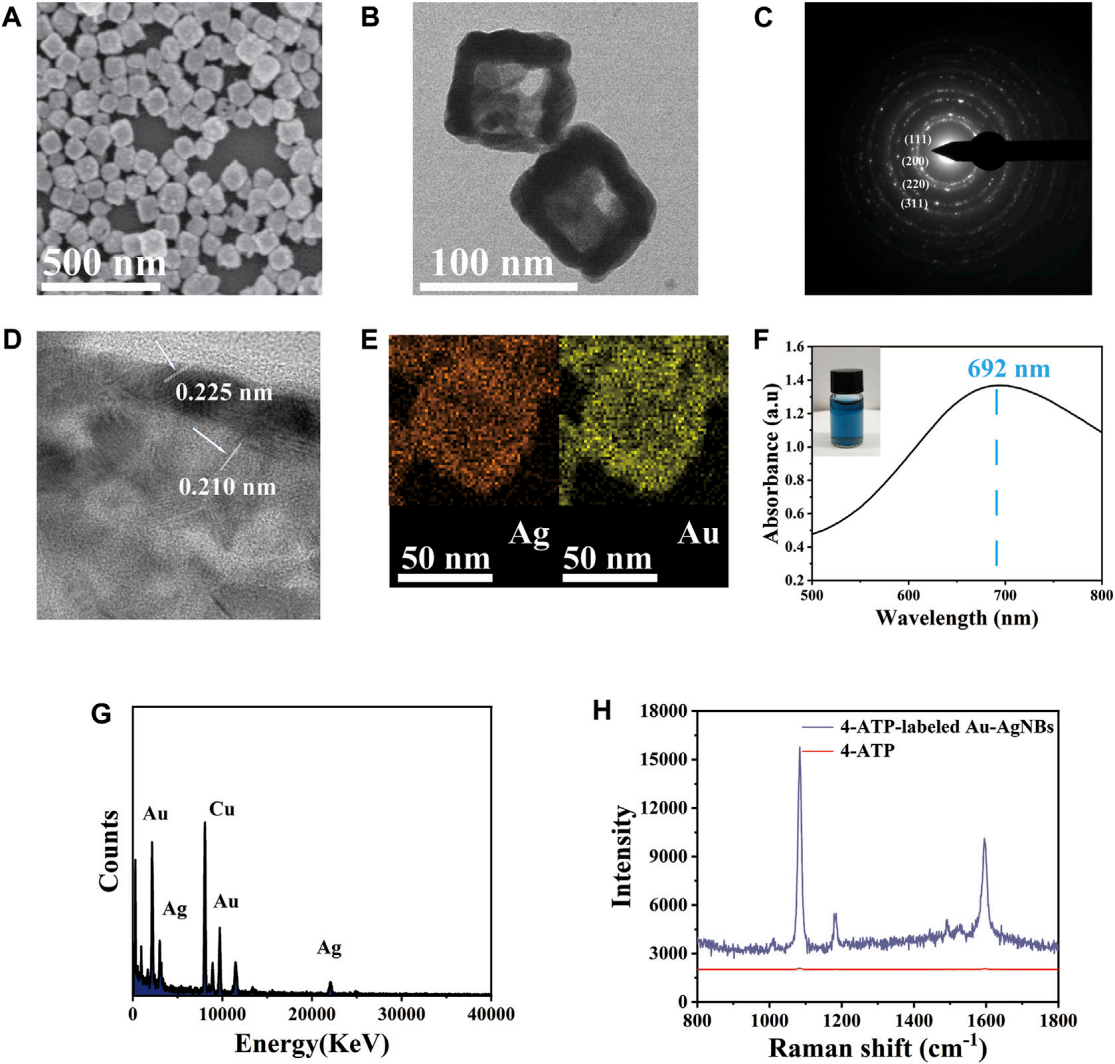
The representative structure of Au-AgNBs was generated in one step. **(A)** SEM of Au-AgNBs, **(B)** TEM, **(C)** SAED image, **(D)** HRTEM and EDX mapping **(E)** for Ag element and Au element image, **(F)** UV-Vis-NIR absorption spectrum of Au-AgNBs. **(G)** EDX spectra of Au-AgNBs. **(H)** SERS spectra of 4-ATP and 4-ATP-labeled Au-AgNBs.

Au-AgNBs is a new type of nanomaterial, which has the advantages of high hotspot density and good stability. TEM and SEM were used to characterize the structure and size of Au-AgNBs. The SEM image of Au-AgNBs could be seen in Figure 1A, which indicated that a large amount of Au-AgNBs could be obtained by one-step process with uniform size and good dispersion. It could be seen that in the TEM image, the hollow inner wall and outer wall of Au-AgNBs were obviously different (Figure 1B). The mean side length of Au-AgNBs was 70 nm and the thickness of the wall was 5 nm. As shown in Figure 1C, the four bright rings {111}, {200}, {220}, and {311} indicated that Au-AgNBs were polycrystalline (Gomez-Grana et al., 2013). Usually, nanocages were composed of bimetals and almost all hollow nanostructures needed to use templates to form nanocages (Zhang et al., 2010). The template was often silver nanocubes. In particular, Cl^−^ promoted the formation of silver cube templates through the cap effect, which was consistent with the halide selective stabilization of the {100} surface of Au-AgNBs, while the silver nanocrystals were oxidized to form Au-AgNBs in the inner pore wall (Huang et al., 2009). Figure 1D indicated the plane distances between the tip crystal faces of the inner and outer walls of Au-AgNBs were 0.210 nm and 0.225 nm respectively. The basic diagram of energy dispersive X-ray energy spectrum (EDX) of Au-AgNBs could be seen from Figure 1E, it could be seen that the outer wall composition of Au-AgNBs were dominated by silver and gold. Figure 1F showed the UV-visible spectrum of Au-AgNBs with a broadband maximum of 692 nm, which indicated that a large amount of Au-AgNBs had been prepared. The physical drawing showed the picture of the Au-AgNBs solution in visible blue color. Figure 1G was the energy dispersive X-ray spectrum (EDX) of Au-AgNBs. It showed that Au-AgNBs is mainly composed of gold and silver and the peak of copper in the electron spectrum was mainly due to the use of copper mesh as the test substrate. The Raman spectra of 4-ATP and 4-ATP-labeled Au-AgNBs were shown in Figure 1H. 4-ATP and Au-AgNBs were connected to each other mainly through Au-S bonds (Jang and Keng, 2008). As could be seen from the figure, the Raman spectrum of 4-ATP showed that the SERS signal was very weak. In contrast, significantly enhanced SERS signal could be observed for 4-ATP-labeled Au-AgNBs indicating that Au-AgNBs had a strong SERS effect. The analytical enhancement factor (EF) of Au-AgNBs was calculated as EF=(I_SERS_/C_SERS_)/(I_RS_/C_RS_) (Mahmoud and El-Sayed, 2010). SERS and RS represent SERS condition and non-SERS condition, while C and I represent the concentration and intensity, respectively. When C_SERS_ = 1 × 10^−6^ M, C_RS_ = 10^−1^ M and the intensity measured at 1,081 cm^-1^, the EF calculated was 7.531×10^5^. This effect was due to the coupling of its inner and outer surfaces, leading to strong optical absorption and high SERS enhancement. Therefore, compared with the ordinary noble metal substrate, the Au-AgNBs array had significant SERS signal enhancement ability.

### Characterization of Au-AgNBs array

**FIGURE 2:**
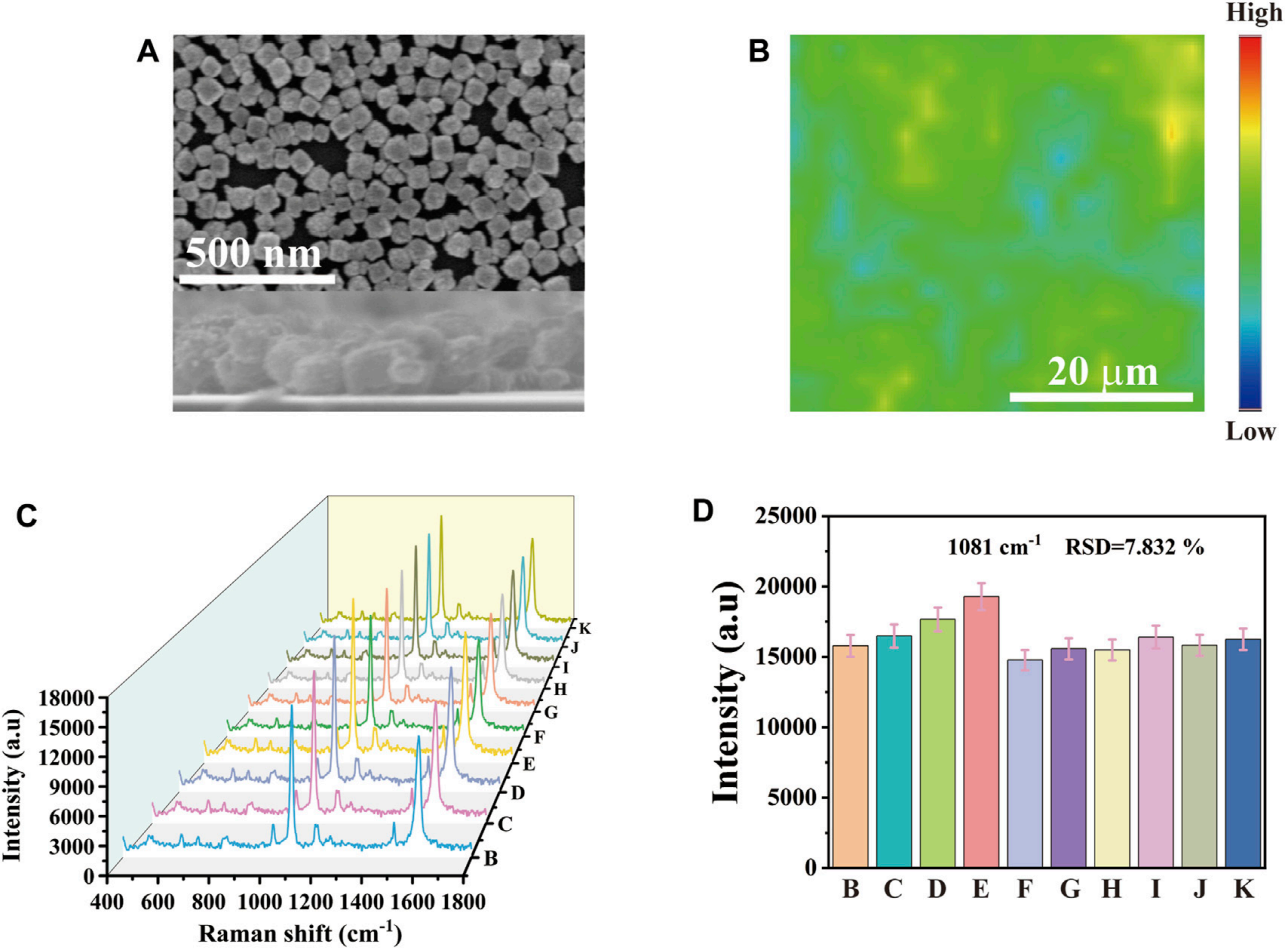
**(A)** SEM of cross section and plane for monolayer Au-AgNBs array. **(B)** SERS mapping of Au-AgNBs array at 1,081 cm^−1^. **(C)** SERS spectra with a peak intensity of 1,081 cm^−1^ were obtained from 10 randomly selected points within the 40 × 40 μm^2^ region of the substrate of the Au-AgNBs array and **(D)** the histogram of spectral intensity at 1,081 cm^−1^.

Excellent SERS substrate performance is the key to practical application. As shown in Figure 2A, the SEM image showed the side view of Au-AgNBs array. It could be seen that Au-AgNBs was highly uniform and orderly arranged, with an average height of about 70 nm. 40 × 40 μm^2^ area was randomly selected on the array marked with 4-ATP for SERS intensity mapping measurement. Each pixel in the spatial position of the mapping image represented the signal strength at 1,081 cm^-1^. These signals were related to the distribution of Au-AgNBs on the array surface. Although there were a few blue and yellow areas in the image, most areas show relatively stable green, indicating that the SERS substrate had a high uniformity as shown in Figure 2B. In order to more intuitively verify the uniformity of the detection substrate, 10 random points were selected on the substrate surface for SERS spectrum measurement. Figure 2C showed the SERS spectrum of the random points. It could be seen that the signal strengths of the selected points were relatively consistent. The histogram in Figure 2D intuitively showed the slight fluctuation of the spectrum and its relative standard deviation (RSD) was 7.832%, indicating that the Au-AgNBs array had good signal uniformity.

### Optimization of parameters

**FIGURE 3:**
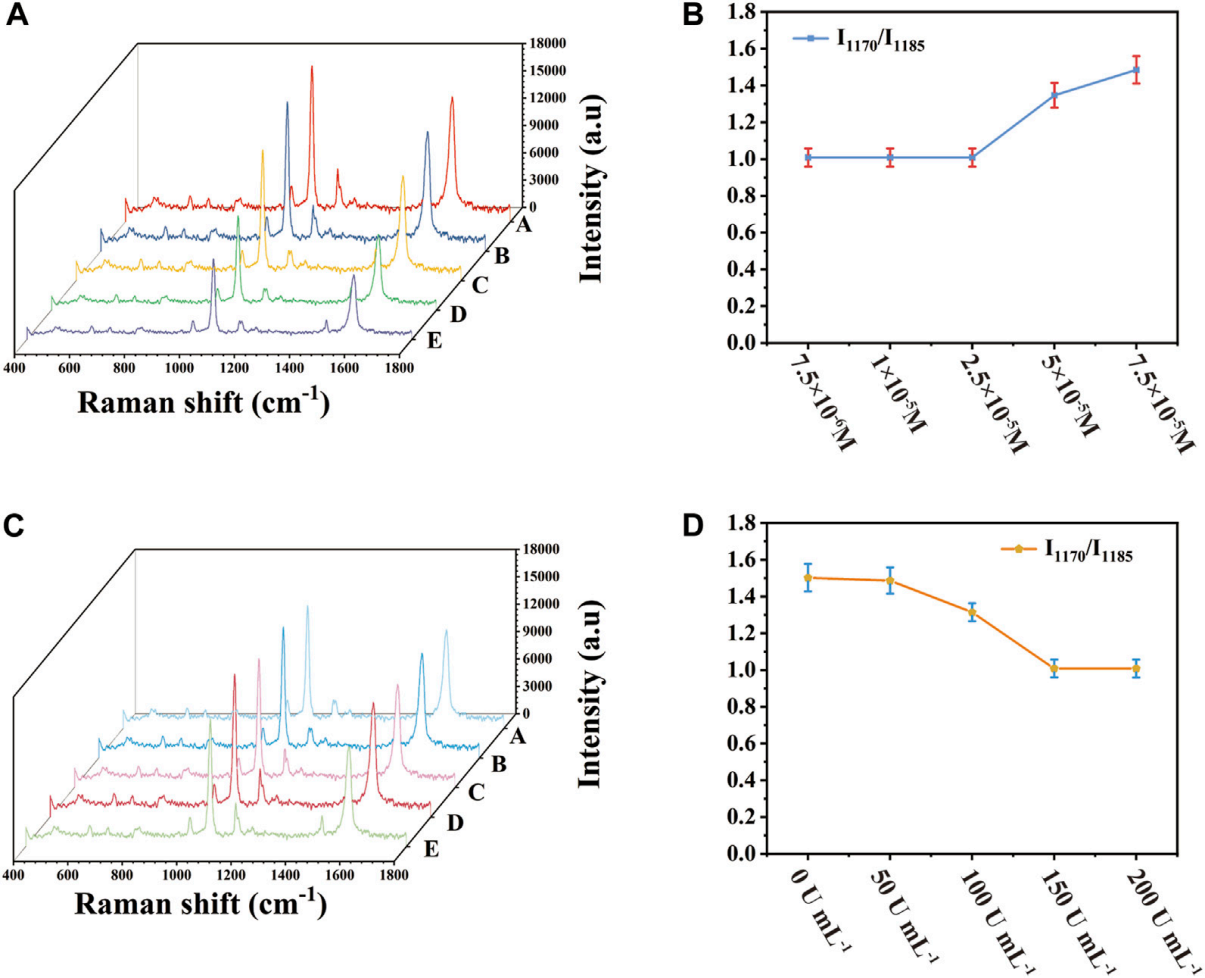
Relationship between 4-ATP concentration and SOD concentration and I_1170_/I_1185_ **(A)** SERS spectrum when 4-ATP concentration was changed, **(B)** corresponding scatter plot, **(C)** SERS spectrum when SOD concentration was changed, **(D)** corresponding scatter plot.

The concentration of 4-ATP and SOD had a strong influence on the detection sensitivity. By optimizing these parameters, the sensitivity of SERS detection platform could be further improved. When 4-ATP was adsorbed on the surface of Au-AgNBs, under the irradiation of laser, 4-ATP would be oxidized into DMAB. In the presence of SOD, SOD could combine with OH^−^ in solution to remove oxide, thus inhibiting the formation of DMAB and then SERS signal showed the characteristic peak of 4-ATP. The value of I_1170_/I_1185_ was selected as the index parameter of the optimization result, the detection sensitivity was positively correlated with I_1170_/I_1185_. In the process of preparing SERS substrate, 4-ATP with different concentrations was added and then SOD solution with the same solubility of 150 U mL^−1^ was dripped respectively. As shown in Figure 3A, the peak intensity of I_1170_ gradually increased with the increase of 4-ATP concentration. It was shown in Figure 3B that when the 4-ATP concentration was 2.5 × 10^−5^ M, the ratio of I_1170_/I_1185_ was close to 1, and I_1170_/I_1185_ ratio remained unchanged with the increase of 4-ATP concentration. Therefore, when the SOD concentration was 150 U mL^-1^, the optimal 4-ATP concentration was 2.5 × 10^−5^ M. Similarly, by controlling the concentration of 4-ATP to 2.5 × 10^−5^ M and changing the concentration of SOD from 0 U mL^−1^–150 U mL^−1^, as shown in Figures 3C, D, the ratio of I_1170_/I_1185_ gradually decreased with the increase of SOD concentration. When SOD concentration was 150 U mL^-1^, the ratio of I_1170_/I_1185_ was close to 1 and when SOD concentration was increased again, the ratio of I_1170_/I_1185_ hardly changed, indicating that when SOD concentration was 150 U mL^−1^, the conversion of 4-ATP could be completely inhibited. Therefore, SOD with the concentration of 150 U mL^−1^ and 4-ATP with the concentration of 2.5 × 10^−5^ M were selected as the best concentrations.

### Characterization of the sensor performance

**FIGURE 4:**
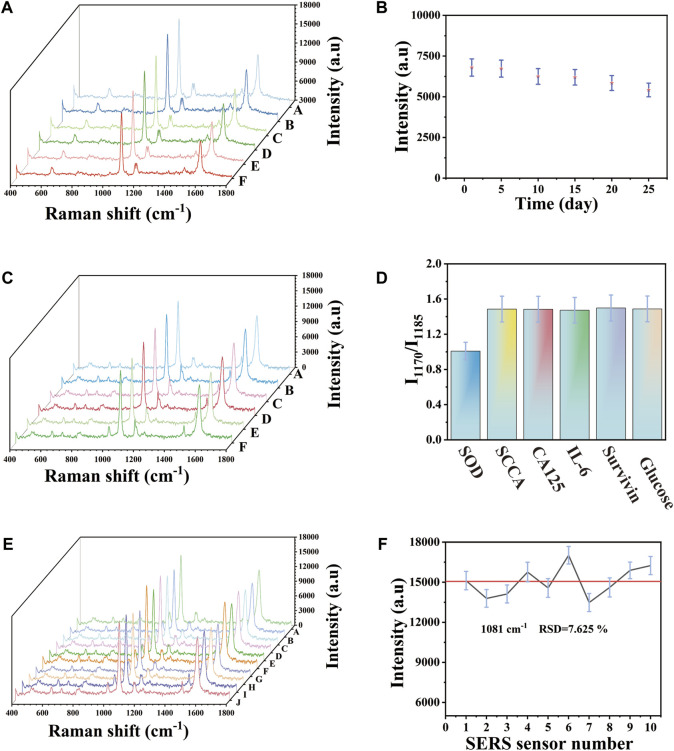
**(A)** SERS spectra of 4-ATP-labeled Au-AgNBs array stored for different days. **(B)** The scatter graph corresponding to SERS intensity at 1,081 cm^−1^. Specificity based on Au-AgNBs array. **(C)** SERS spectra of analytes (1) SOD, (2) SCCA, (3) CA125, (4) IL-6, (5) survivin, (6) glucose. **(B)** Histogram corresponding to I_1170_/I_1185_. Reproducibility of Au-AgNBs array. **(E)** SERS spectrum at 1,081 cm^−1^. **(F)** Scatter diagram of peak intensity at 1,081 cm^−1^.

The stability of SERS substrate was evaluated. The prepared SERS substrate was stored in a sealed container at 20°C and SERS detection was taken on the SERS substrate at the 0, 5, 10, 15, 20, and 25 days respectively. As can be seen in Figure 4A, there was no significant difference in the SERS spectrum peak and spectrum shape. Figure 4B showed the corresponding scatter plot, with the peak intensity of 1,081 cm^−1^ as the characteristic peak and the peak intensity of the 15th day was 8.803% lower than that of the 0 day. Among them, the peak intensity on the 25th day was still maintained at 80% of the initial intensity compared with the peak intensity on the 0 day, indicating that the SERS array base had stable SERS enhanced effect and storage stability. As we all know, the selectivity of SERS platform was of great significance in the actual analysis of biological samples. In order to evaluate the selectivity of SERS immune substrate. The substances were selected that may exist in the detection environment as interfering substances and detected specific markers SOD and non-specific biomarkers or proteins (SCCA, CA125, IL-6, survivin, glucose) of the same concentration (100 U mL^−1^) in PBS buffer. I_1170_/I_1185_ was selected as the tracer of SOD. As shown in the spectrum in Figure 4C, the peak intensity of I_1170_ of SOD was similar to that of I_1185_, while the peak intensity of I_1170_ of other substances was obviously higher than that of I_1185_. The histogram of Figure 4D indicated the results more clearly. When SOD existed, the I_1170_/I_1185_ ratio was obviously lower than that of the solution without SOD. Under the above optimal conditions, the reproducibility of another important parameter of the SERS platform was studied. According to Figure 4E, the SERS spectra was studied by selecting of ten independent experiments conducted at different times. There was almost no difference between these SERS spectra. The broken line graph of the SERS spectrum was shown in Figure 4F. With 1,081 cm^−1^ as the characteristic peak, the peak intensity deviation was 7.625%. This small change showed that the SERS platform had good reproducibility. In order to study the differences between different batches of Au-AgNBs arrays, the Au-AgNBs arrays made at different batches were compared. Figure S1A showed the differences of SERS spectrum. The Au-AgNBs array marked with 4-ATP prepared at different batches were detected by SERS. As shown in Figure S1B, with 1,081 cm^-1^ as the reference peak, the intensity deviation of the four peaks was small (2.407%), indicating that there was almost no difference between Au-AgNBs array prepared at different times.

### Application of detection platform in serum

**FIGURE 5:**
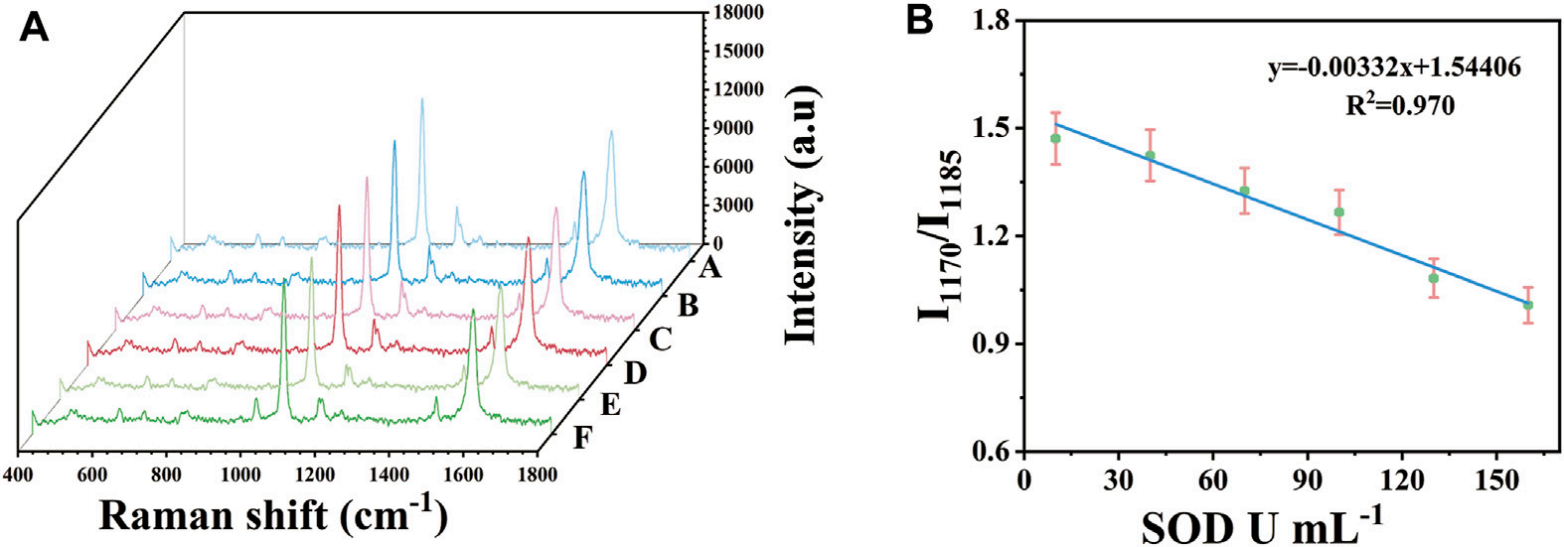
After applying different concentrations of SOD in serum (10 U mL^−1^–160 U mL^−1^), obtained SERS spectrum **(A)** and calibration curve **(B)**.

Under the above optimization conditions, the capability of SERS platform for rapid analysis of SOD was evaluated. In order to combine the immunosensor with practical application, it was used to detect the concentration of SOD in serum. Au-AgNBs ordered arrays with the size of 0.8 × 0.8 cm^2^ which adsorbed 4-ATP on their surfaces according to the detection principle were adjusted to the PH = 9. The SOD solution was diluted in the purpose-made serum (without SOD) to the concentration of 10 U mL^−1^, 40 U mL^−1^, 70 U mL^−1^, 100 U mL^−1^, 130 U mL^−1^ and 160 U mL^−1^ respectively. 20 μL of the above solutions was dropped on different SERS detection platforms, then the samples were continuously irradiated under Raman microscope. The SERS spectrum were continuously measured for 1 s in a step of 1 min until the spectral shape did not change. 10 points were selected randomly of the detection platform for measurement and the average SERS spectrum were calculated for quantitative detection of SOD in the sample solution. Figure 5A presented the SERS spectra of SOD solutions with different concentrations. It was obvious that with the increase of SOD concentration, the process of 4-ATP converting to DMAB was gradually inhibited, which was reflected in the gradual decrease of the ratio of I_1170_/I_1185_. By using the ratio of I_1170_/I_1185_ as parameter, a linear calibration chart for quantitative assessment of SOD concentration was constructed as shown in Figure 5B. When the SOD concentration was from 10 U mL^−1^–160 U mL^−1^, the ratio of I_1170_/I_1185_ was almost linearly related to the concentration of SOD. Its linear regression equation was y = −0.00332x+1.54406 and the relative coefficient (*R* ^2^) was 0.970. The limit of quantitation (LOQ) of the SERS platform for SOD was 10 U mL^−1^. It showed that the SERS platform had a good linear relationship at the concentration range from 10 U mL^−1^–160 U mL^−1^, which could realize SERS detection of SOD activity in serum.

### Clinical serum samples analysis

**FIGURE 6:**
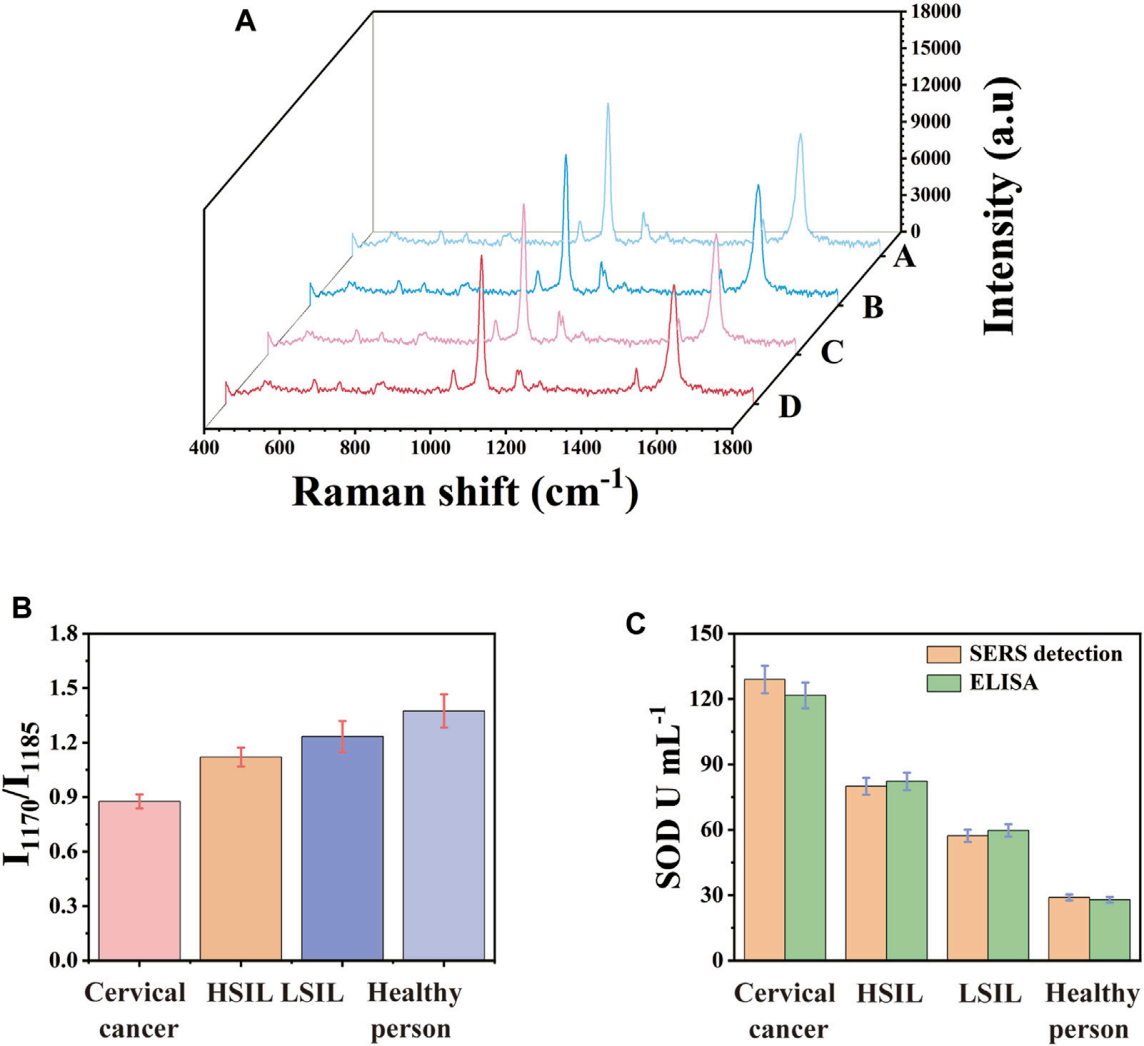
**(A)** Mean SERS spectra of clinical samples. **(B)** I_1170_/I_1185_ histogram of clinical samples, **(C)** comparison histogram of SOD concentration in clinical samples detected by ELISA and SERS.

SERS platform was used to analyse SOD quantitatively to prove the accuracy, reliability and clinical practicability of the analysis. Clinical serum samples of healthy people, LSIL, HSIL and the cervical cancer were studied. By calculating the ratio of I_1170_/I_1185_ in SERS spectrum, the concentration difference of SOD in serum of different populations could be calculated. The used clinical serum samples needed not to be diluted and they were directly used as sample solution. Each sample was measured three times. 10 random test points were measured on the surface of the detection platform each time. Every spectra was the average result of 30 different serum samples. These results are analyzed and the standard deviation is calculated. Figure 6A showed the mean SERS spectrum of clinical samples. It could be seen that as the disease progresses, the characteristic peak of SERS spectrum gradually changed from 4-ATP to DMAB, which was the ratio of I_1170_/I_1185_ gradually increasing. The concentration of SOD in each actual sample was determined by fitting I_1170_/I_1185_ into the linear regression equation of the calibration curve. The concentration of SOD in the serum of patients with cervical cancer was significantly lower than that of HSIL, LSIL and normal people. Figure 6B directly showed the ratio difference of I_1170_/I_1185_ in SERS images. As a detection method for biomarkers, ELISA was used to detect the actual samples and calculated the average concentration. Figure 6C allowed a more intuitive comparison of the SOD concentration in the actual samples detected by ELISA kit and SERS platform. As shown in Table 2, the average concentration of SOD in the serum of healthy people, LSIL, HSIL, and the cervical cancer detected by SERS platform were 129.1 U mL^−1^, 79.95 U mL^−1^, 57.24 U mL^−1^, and 28.96 U mL^−1^, respectively. The concentration of SOD detected by ELISA were 121.7 U mL^−1^, 82.19 U mL^−1^, 59.72 U mL^−1^ and 27.88 U mL^−1^ respectively and the relative errors of the two methods were −3.87%, 4.1%, 2.73% and −6.08% respectively. The results showed that there was no significant difference between SERS platform and ELISA detection results, which confirmed that the SERS platform could be used for early clinical screening of cervical cancer.

**TABLE 2:** Serum and ELISA immunoassay results from clinical serum samples.

Sample	SERS sensor (U mL^−1^) (mean)	ELISA (U mL^-1^) (mean)	Relative error [%]
Cervical cancer	28.96	27.88	−3.87
HSIL	57.24	59.72	4.15
LSIL	79.95	82.19	2.73
Healthy person	129.1	121.7	−6.08

## Conclusion

In this work, Au-AgNBs were orderly arranged on the substrate by the method of oil-water interface self-assembly and a SERS platform capable of quantitative detection of SOD was successfully constructed by the changes of Raman signal molecular characteristic peaks. The experimental results showed that the prepared SERS platform had good performance in homogeneity, reproducibility, selectivity and stability. When the concentration was from 10 U mL^−1^–160 U mL^−1^, the platform could quantitatively detect SOD in human serum and the LOQ was 10 U mL^−1^. The clinical application research of its detection ability was carried out. The SERS platform was used to detect clinical serum samples of healthy people, LSIL, HSIL and cervical cancer patients. In the clinical detection of SOD, turbidimetry and electron spin resonance (ESR) spectroscopy took a long time and the cost was expensive. At the same time, they had low sensitivity and needed to buy special large instruments. On the contrary, the SERS platform had shorter detection time, the entire test could be completed in 20 min and it also had higher sensitivity, simple operation and low price. The detection results of SERS were consistent with ELISA, indicating that it has broad application prospects in early diagnosis of cervical cancer.

## Data Availability

The original contributions presented in the study are included in the article/Supplementary Material, further inquiries can be directed to the corresponding author.
