# Supplementary material for: Rapid and sensitive detection of superoxide dismutase in serum of the cervical cancer by 4-aminothiophenol-functionalized bimetallic Au-Ag nanoboxs array
Source: Front Bioeng Biotechnol. 2023 Mar 2;11:1111866. doi: 10.3389/fbioe.2023.1111866 (PMC10032346; doi:10.3389/fbioe.2023.1111866)
Supplement: Supplementary file 1 [file DataSheet1.doc]

**Supporting Information**

**Rapid and sensitive detection of superoxide dismutase in serum of the cervical cancer by 4-aminothiophenol-functionalized bimetallic Au-Ag nanoboxs array**

Ji Xia^a1^, Gao-Yang Chen^b1^, You You Li^a^, Lu Chen^a^ and Dan Lu^ac*^

^a^Institute of Translational Medicine, Medical College, Yangzhou University, Yangzhou, PR China

^b^Department of Oncology, The second People's Hospital of Taizhou City

^c^Jiangsu Key laboratory of integrated traditional Chinese and Western Medicine for prevention and treatment of Senile Diseases, Yangzhou University, Yangzhou, 225001, PR China

*E-mail: ludan1968@126.com

**The Difference of the SERS Au-AgNBs array**

In order to study the differences between different batches of Au-AgNBs arrays, the Au-AgNBs arrays made at different batches were compared. Figure S1A showed the differences of SERS spectrum. The Au-AgNBs array marked with 4-ATP prepared at different batches were detected by SERS. As shown in Figure S1B, with 1081cm^-1^ as the reference peak, the intensity deviation of the four peaks was small (2.407 %), indicating that there was almost no difference between Au-AgNBs array prepared at different times.


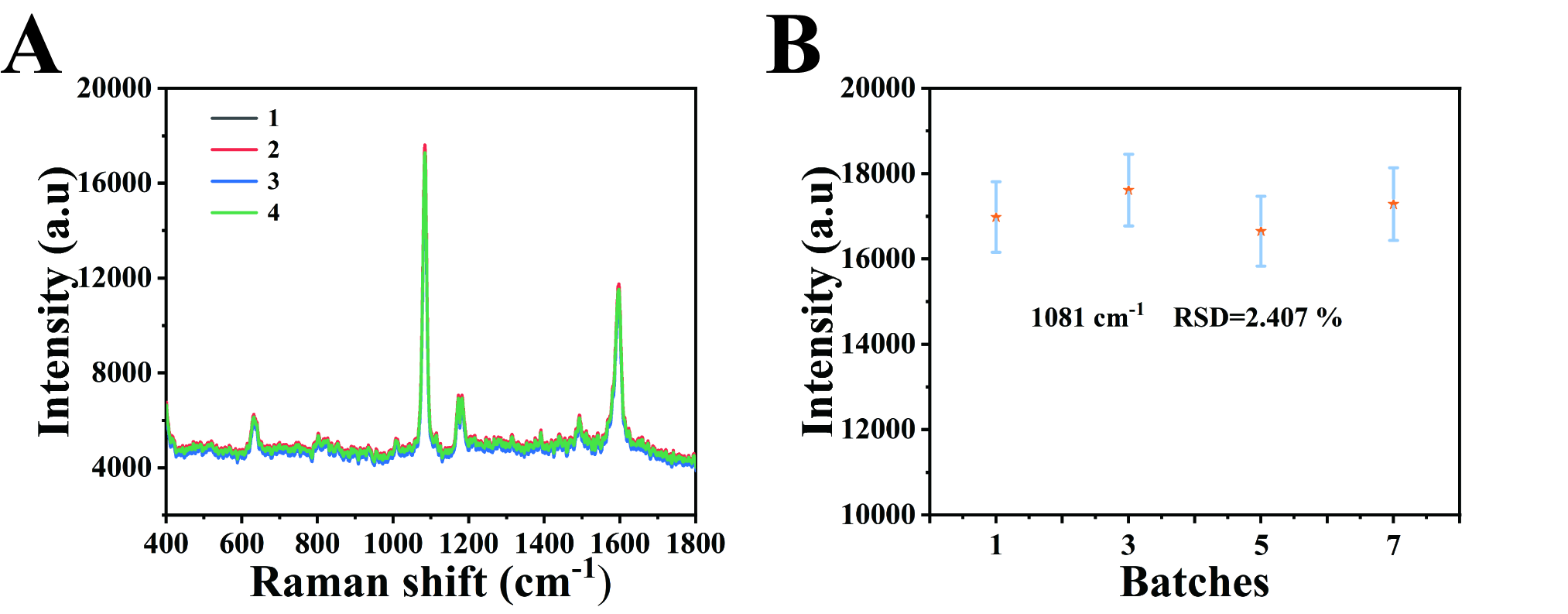

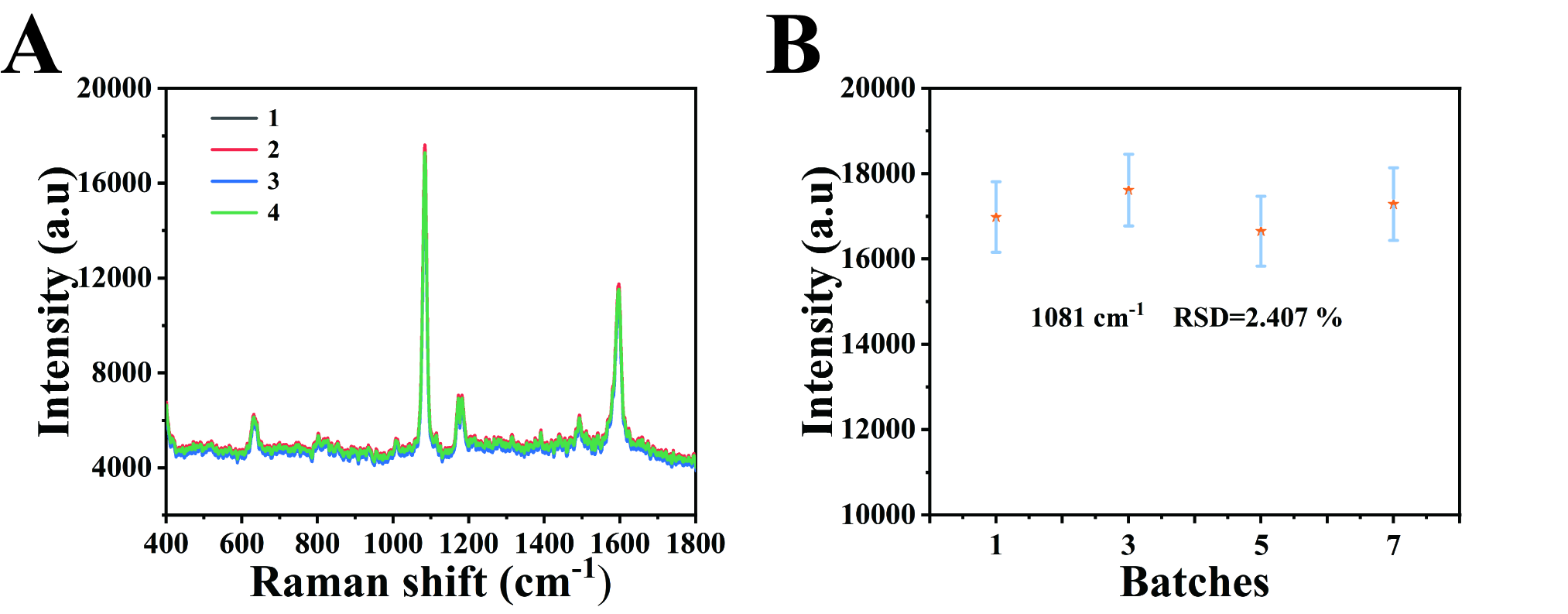


**FIGURE S1** (A) The difference of the Au-AgNBs array labeled by 4-ATP. (B) SERS intensities at 1081 cm^-1^ of Au-AgNBs array for different batches.

**TABLE S1** The characteristic bands of 4-ATP and DMAB

| Groups | Characteristic bands (cm^-1^) | | | |
| --- | --- | --- | --- | --- |
| 4-ATP | 1081 | 1170 | 1392 | 1442 |
| DMAB | 1081 | 1185 | 1489 | 1590 |

SERS detection was performed on the 4-ATP labeled Au-AgNBs array with the detection range of 200-1800 cm^-1^. As shown in the Raman spectrum (Figure S1), it could be seen that the characteristic peak of Au-S bond vibration was 257 cm^-1^ (Supporting Information).


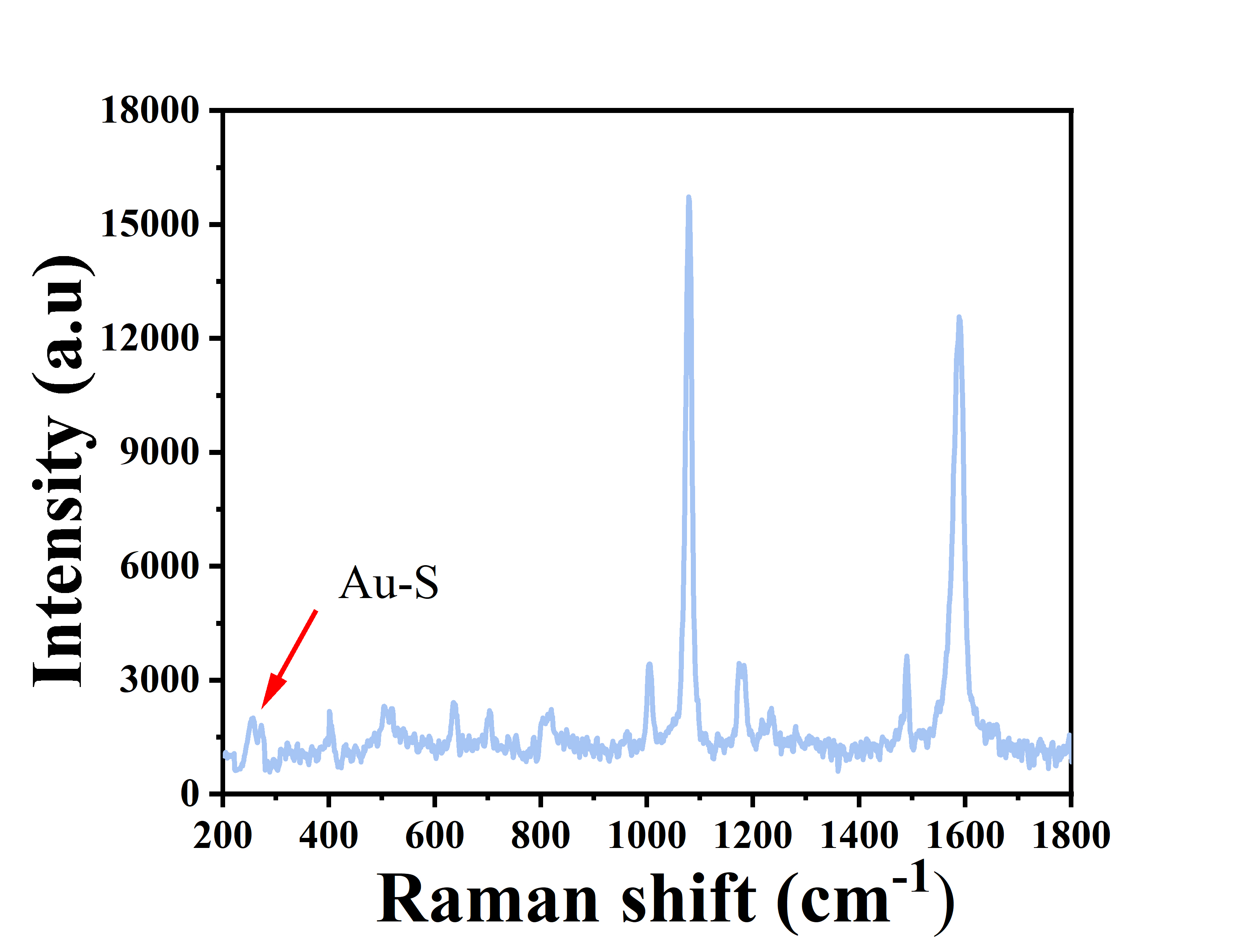


FIGURE S2 The Raman spectrum of 4-ATP labeled Au-AgNBs array at the range of 200-1800 cm^-1^.
